# Reply to Wang et al.: Linking microbial metabolites and gut epithelial responses to microbiome-directed therapeutic foods

**DOI:** 10.1073/pnas.2613406123

**Published:** 2026-06-08

**Authors:** Yi Wang, Hao-Wei Chang, Michael J. Barratt, Jeffrey I. Gordon

**Affiliations:** ^a^ The Edison Family Center for Genome Sciences and Systems Biology, Washington University School of Medicine, St. Louis, MO 63110; ^b^Newman Center for Gut Microbiome and Nutrition Research, Washington University School of Medicine, St. Louis, MO 63110; ^c^Department of Pathology and Immunology, Washington University School of Medicine, St. Louis, MO 63110

We thank Wang and colleagues for their thoughtful comments. We agree that butyrate is a plausible but likely not the only metabolite that contributes to the effects of MDCF-2-induced microbiome repair on gut epithelial cell biology (1, 2). Current evidence supports the view that MDCF-2–mediated microbiome repair is a complex process initiated by metabolism of its components, including certain of its glycan structures, by a subset of gut microbial community members (3, 4). The resulting products, whether derived directly from these components or from downstream metabolic pathways, may either directly signal to the host, or impact the metabolism of other organisms in the community. Furthermore, the effects of the products of this microbial commerce need to be contextualized based on the physical locations of the organisms relative to one another and host cells. In addition, cells in the perpetually renewing intestinal epithelium should be viewed as a function of their state of differentiation as well as their cellular “neighborhoods.”

In Wang et al., we described one approach for “reverse translation” studies designed to gain deeper mechanistic understanding of children’s responses to MDCF-2 treatment; namely, introducing intact uncultured microbial communities from carefully phenotyped participants in randomized controlled clinical trials of MDCF-2 into gnotobiotic mouse models that allow features of the trial to be re-enacted (1). Another approach is to use defined consortia of cultured, genome-sequenced members of the microbial communities of the very population where the clinical trials are performed. Membership of these consortia can be manipulated prior to colonization of the gnotobiotic mice as can be their diets, allowing systematic testing of MDCF-2 components (4–6). Datasets generated from single nucleus RNA sequencing of different regions of the intestine and spatial transcriptomics of the same regions can be integrated using various algorithms (e.g., refs. 7–9); the results should yield higher-resolution views of the transcriptional responses of various cell types (e.g., tuft and goblet cell) to dietary components as a function of their cellular neighborhood, and help identify signaling/metabolic pathways whose expression is regulated by microbiome-directed therapeutic agents. These datasets, in turn, could be used to direct hypothesis-driven targeted mass spectrometry efforts that seek candidate microbial effectors of host biology. The hope is that these candidate effectors, once identified and synthesized, can be tested for their pharmacologic effects (e.g., using organoids or gnotobiotic animals).

These bioactive compounds are products of “master microbial chemists” who have learned how to manipulate our biology, and represent part of the shared dream of this field to populate our 21st century medicine cabinets with new therapeutic agents. All of this underscores the complexity and dynamism of the gut microbiome and its habitat, and the need for a sense of awe, combined with persistence, on the part of investigators seeking to understand its contributions to health and disease.
